# Pyrolysis and Steam Reforming in Series of Footwear Waste to Hydrogen-Rich Syngas and Char for CO_2_ Adsorption

**DOI:** 10.3390/molecules31193490

**Published:** 2026-10-01

**Authors:** Lucas David Biondo, Luiz Gustavo Tyska, Cezar de Mattos, Daniele Perondi, Marcelo Godinho

**Affiliations:** 1 Postgraduate Program in Engineering Processes and Technology, University of Caxias do Sul—UCS, Caxias do Sul 95070-560, Brazil; ldbiondo@ucs.br (L.D.B.); lgtyska@ucs.br (L.G.T.); cmattos@ucs.br (C.d.M.); dperondi1@ucs.br (D.P.); 2 UCSGRAPHENE Research and Development Unit, University of Caxias do Sul—UCS, Caxias do Sul 95070-560, Brazil

**Keywords:** footwear waste, pyrolysis, steam reforming, hydrogen production, carbon dioxide capture

## Abstract

Footwear manufacturing generates large volumes of heterogeneous polymeric waste. In this context, thermochemical routes offer a pathway to convert this waste into hydrogen-rich syngas and value-added solid products (char). This study presents a proof-of-concept for the integrated valorization of mixed footwear waste via pyrolysis and steam reforming in series for hydrogen production and waste valorization. Footwear waste was pyrolyzed and the resulting volatiles were steam-reformed in a two-stage system, while reforming temperature (700, 800, and 900 °C) and steam-to-carbon ratio (2.5, 7, and 12) were assessed as quantitative factors; hydrogen (H_2_) yield was adopted as the primary response variable, while CO, CO_2_, CH_4_, and light hydrocarbons (C_2_–C_4_) were monitored as complementary responses to assess gas quality, and the solid char was characterized in terms of textural properties and CO_2_ uptake. The highest hydrogen yield, reaching 16.42 mmol g^−1^, was obtained at a reforming temperature of 900 °C combined with a steam-to-carbon ratio of 7, whereas char produced by pyrolysis at 600 °C exhibited a specific surface area (S_BET_) of reaching 86.47 m^2^ g^−1^ and a CO_2_ adsorption capacity of reaching 20.13 mg g^−1^. These findings indicate that pyrolysis coupled with steam reforming can convert footwear waste into a hydrogen-rich gas while simultaneously yielding a char with adsorptive potential, suggesting a feasible route to reduce the environmental impact.

## 1. Introduction

The footwear industry relies on a wide range of material diversity to achieve mechanical strength, flexibility, comfort, durability, and aesthetic properties. However, this material diversity also creates a substantial end-of-life management challenge [1,2]. Footwear wastes, including manufacturing residues and post-consumer waste, are typically heterogeneous and difficult to separate economically [3,4]. Mechanical recycling routes are most effective for homogeneous footwear waste; in contrast, polymer blends along with material diversity limit the quality and applicability of recycled products [5,6]. Additionally, even a homogeneous fraction can find limits to be used as an equivalent material and is used as a filler in polymeric composites [7].

Thermochemical conversion offers a complementary route for the valorization of complex and difficult-to-recycle wastes. Previous characterization studies have shown that footwear waste can contain materials with appreciable energy content [8]. In particular, pyrolysis can convert wastes in an oxygen-free or oxygen-deficient atmosphere into a solid (char) and a volatile fraction. The resulting char may be valorized as a carbonaceous material, and the volatiles may be further upgraded to hydrogen-rich syngas.

A promising strategy for upgrading pyrolysis volatiles is steam reforming [9] in a two-stage pyrolysis–steam-reforming configuration. Pyrolysis volatiles are subsequently contacted with steam at elevated temperature, promoting reactions that convert hydrocarbons and tar compounds into an expected hydrogen-rich gas. Two-stage processes have been widely investigated for waste plastics, showing their potential to enhance hydrogen and syngas production relative to pyrolysis alone [10].

Among the parameters governing steam reforming performance, reforming temperature and the steam-to-carbon ratio are particularly important. The effect of these variables depends on the nature of the pyrolysis volatiles, which is itself strongly influenced by the feedstock.

Increasing temperature generally favors the endothermic reforming of hydrocarbons and may improve the conversion of methane and heavier hydrocarbons. Similarly, steam addition can promote reforming and water-gas shift (WGS) reactions [11].

In addition, rather than treating char produced in pyrolysis as a low-value by-product, its potential use in carbon capture should be considered. Their adsorption performance is influenced by the original feedstock and pyrolysis conditions, particularly temperature, which affects the carbonization degree, surface chemistry, and pore development [12].

In this study, the still-scarce integrated valorization of realistic mixed footwear waste was assessed as a proof-of-concept. In order to provide a consistent volatile feed to the steam reforming stage, the experiments were conducted at 600 °C in the pyrolysis stage. The influence of reforming temperature (700, 800, and 900 °C) and steam-to-carbon ratio (2.5, 7, and 12) was investigated, and the CO_2_-capture potential of the resulting char was assessed.

## 2. Results

### 2.1. Footwear Waste Characteristics

The realistic footwear waste investigated in this study is composed predominantly of polyesters (~65%) > polyurethane (PU) (~25%) > ethylene-vinyl acetate (EVA) (~6%) > cotton (~4%). A representative image of the material is provided in Figure S1 (Supplementary Materials).

The proximate analysis results of the footwear waste used in this study (Table 1) reveal a material with low moisture content (0.35%), very close to that observed for polyethylene terephthalate (PET) (0.36%), and markedly lower than the other expected components, such as cotton (3.72–4.56%), cotton/polyester blend (2.52%), and polyurethane (1.79–3%) [13,14,15,16]. Low moisture is desirable for the pyrolysis and steam reforming process, as it reduces energy demand [17]. The volatile matter content (78.6%), in turn, was the lowest among all the compared materials, falling below the typical values of polyester (84.05%), PU (83.9–86.1%), PET (86.1–89.3%), and, most notably, cotton (87.76–96.7%) [13,14,15,16,18]. The fixed carbon content (13.9%) fell within the mid-to-upper range, close to PET (13.4%) and the cotton/polyester blend (13.04%), and higher than that observed for pure cotton samples (2.9–6.9%) and PU (8.3–11.9%) [13,15,16,18]. The most striking feature, however, is the ash content (7.5%), considerably higher than all values reported in the literature (0 to 5.57%), possibly associated with fillers, pigments, and stabilizers typically found in plastic waste mixtures [19]. This high ash content is undesirable for pyrolysis and steam reforming processes, reducing oil and gas yield [17].

Table 1 also presents the ultimate analysis of the footwear waste sample in this study. The carbon content (58.42%) was intermediate between that observed for cotton (41.57%) and the cotton/polyester blend (48.78%), approaching, however, that of polyester (60.15%) [15], which is consistent with the predominance of polyester in the footwear waste used in this study. The hydrogen content (6.63%) was close to the values of cotton (6.02%) and the cotton/polyester blend (5.63%) but considerably higher than that of polyester (4.22%) [15]. The nitrogen content (1.43%) was higher than that found in cotton (0.66%), cotton/polyester blend (0.23%), and polyvinyl chloride (0.14%), although well below the high content observed for polyurethane (4.7%) [14,15,16]. This nitrogen content is consistent with the literature, given that polyurethane is the second most representative component of the sample. Sulfur (0.11%), although low, was present at lower levels than in polyvinyl chloride (0.78%) but higher than in polyester and the cotton/polyester blend, which showed no detectable sulfur [14,15]. Finally, the oxygen content (25.91%, obtained by difference) was significantly lower than that of cotton (49.39%) and the cotton/polyester blend (43.65%) but fell between those of polyester (33.8%) and polyurethane (20.4%) [15,16], the main components of the footwear waste used in this study.

**Figure 1:**
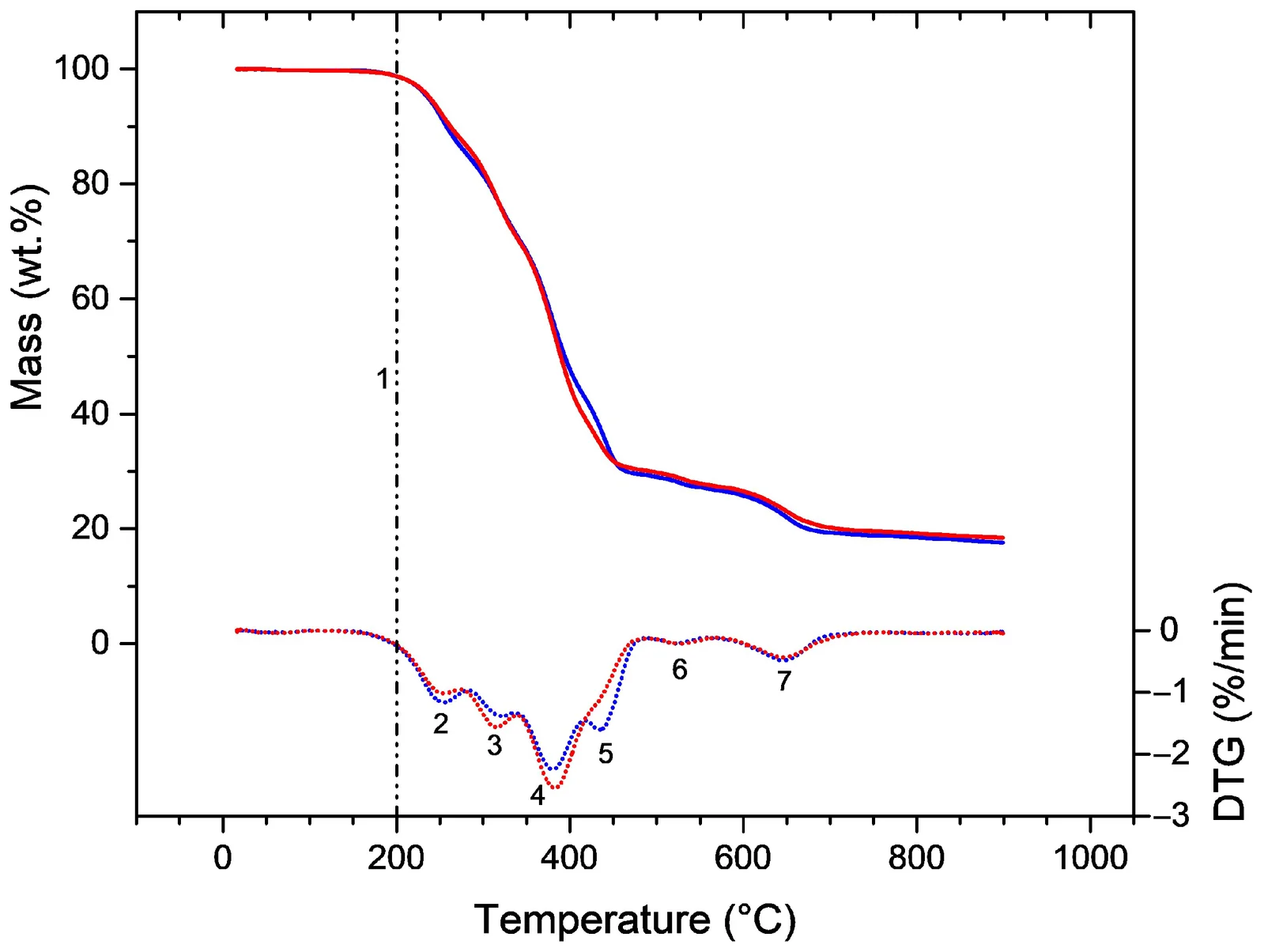
TGA and DTG replicates of footwear waste samples under N_2_.

The two replicate TG/DTG profiles of the footwear waste under an inert atmosphere (N_2_), shown in Figure 1, suggest seven thermal events, preceded by an initial mass loss attributed to moisture release. The first event, at approximately 200 °C, marks the onset of pronounced mass loss; accordingly, water injection into the second stage of the reforming reactor was started at 190 °C in the pyrolysis and steam-reforming runs.

Events 2 and 3, with DTG peaks at approximately 250 and 315 °C, respectively, may receive contributions from the thermal degradation of polyurethane hard segments, whereas events 4 and 5, with DTG peaks at approximately 380 and 430 °C, may receive contributions from the thermal degradation of polyurethane soft segments. This assignment is consistent with [20], who reported that the fraction corresponding to polyurethane hard segments decomposes between 247 and 340 °C and that the soft segments decompose between 340 and 480 °C. Ref. [21], in turn, located hard-segment thermal degradation between 200 and 370 °C and soft-segment degradation between 370 and 500 °C.

Footwear waste is a multicomponent material; the events overlap and may be interpreted as receiving contributions from more than one constituent rather than as isolated peaks. The DTG peak at approximately 380 °C may also receive contributions from cotton. Ref. [22] performed thermogravimetric analysis of pure cotton and reported DTG peaks between 355.4 and 382 °C for heating rates from 5 to 20 °C min^−1^. Events 4 and 5 may likewise include polyester degradation. Ref. [23] analyzed a 100% polyester material by TG and found the onset of the DTG peak at approximately 351 °C and the DTG peak at approximately 428 °C, whereas Ref. [22] reported that the main mass loss of polyester occurred between approximately 381 and 431 °C.

Event 6, with a DTG peak close to 520 °C, is attributed to progressive carbonization of the residue. In the case of EVA, this event coincides with the final stage of main-chain scission of the deacetylated copolymer. The thermal degradation of EVA comprises two steps [24,25]: the first, deacetylation, occurs between 300 and 450 °C and yields a polyunsaturated chain, which may contribute to events 3, 4, and 5; the second, cracking of the polyunsaturated chain formed in the first step, occurs between 450 and 520 °C and may contribute to event 6.

Event 7, with a DTG peak at approximately 640 °C, may be attributed to degradation of the inorganic fraction present in the residue. In plastic mixtures, a mass loss between 600 and 700 °C suggests the presence of thermally stable additives or fillers such as calcium carbonate (CaCO_3_) [26].

### 2.2. Gas Products from Pyrolysis and Steam Reforming in Series of Footwear Waste

The main expected reactions in the pyrolysis and subsequent steam reforming of footwear waste are shown in Table 2.

**Figure 2:**
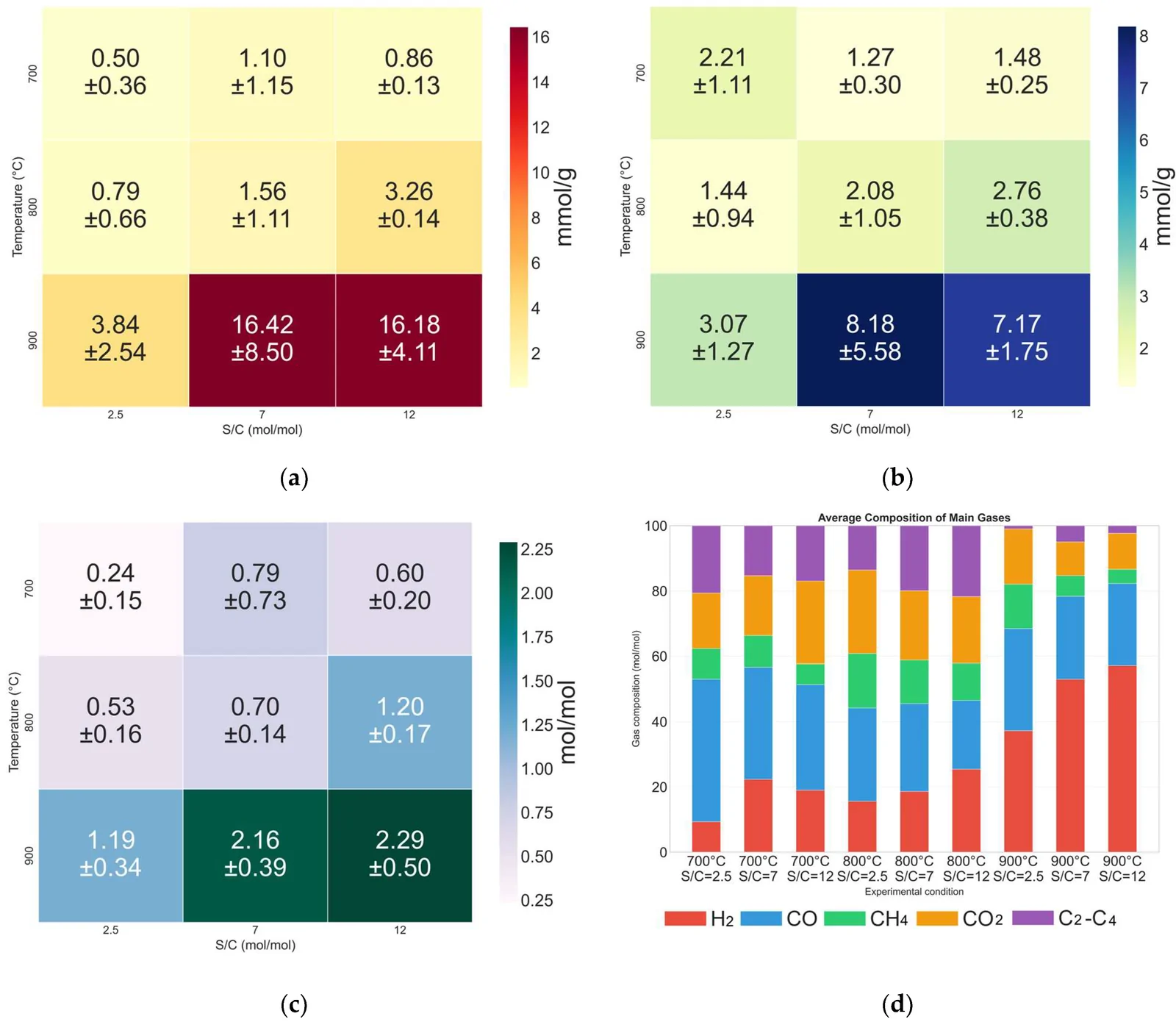
(**a**) Hydrogen yield (H_2_) as a function of the steam reforming temperature and the steam-to-carbon ratio. (**b**) Carbon monoxide yield (CO) under different operating conditions of the reforming process. (**c**) Influence of the process parameters on the H_2_/CO molar ratio of the produced syngas. (**d**) Volumetric chemical composition of the resulting gas (H_2_ and CO fractions) in response to variations in temperature and steam-to-carbon ratio.

The equilibrium of the reactions described in Table 2 is significantly influenced by fundamental process parameters, notably steam reforming temperature and the steam-to-carbon ratio (S/C) [9,27,28,29]. The results regarding the hydrogen and carbon monoxide yields, the H_2_/CO molar ratio, and the overall composition of the produced gas are shown in Figure 2. Therefore, the individual effects of reforming temperature and steam-to-carbon ratio on process efficiency and product gas quality will be discussed in depth. Additionally, a complementary experiment was carried out with pyrolysis-only (R1), heated at 40 °C min^−1^ to a final temperature of 600 °C, in order to analyze the effects of steam reforming. Yields per gram of footwear waste of 0.22 mmol H_2_, 0.33 mmol CO, 1.66 mmol CO_2_, 0.13 mmol CH_4_, and 1.47 mmol C_2_–C_4_ were achieved. A basic mass balance of pyrolysis and pyrolysis coupled with steam reforming experiments is provided in Table S1 (Supplementary Materials).

With a temperature of 700 °C in the reforming stage, an increase in the production of H_2_ (from 0.22 to 0.50–1.10 mmol H_2_ g^−1^) and CO (0.33 to 1.27–2.21 mmol CO g^−1^) is observed in the pyrolysis-only experiment, possibly related to a balance of thermal cracking (R2), reverse WGS (R6), and Boudouard (R7) reactions. The temperature of 700 °C constitutes the lower operating limit of the system and was found to be insensitive to the increase in S/C (Figure 2a,b). At this temperature, H_2_ and CO production remained at a low level, suggesting that 700 °C was below the threshold for activating the steam reforming reactions in the absence of a catalyst and under the other experimental conditions of this study.

At 800 °C, the maintenance of the C_2_–C_4_ content (Figure 2d) suggests equilibrium between thermal cracking (R2) and the reforming reactions (R3, R4, and R5). Accordingly, the H_2_ and CO yields (Figure 2a,b) show only modest increases in this same temperature range, suggesting that no reaction route predominates in the reactor.

The transition from 800 °C to 900 °C changes the scenario with the reduction of the C_2_–C_4_ content (Figure 2d), the reduction in the CH_4_ content, and the increase in H_2_ and CO production, as can be seen in Figure 2a,b. The pronounced reduction in the C_2_–C_4_ content in this range suggests the point at which the reforming reactions (R3, R4) overtake the thermal cracking reactions (R2). This occurrence is consistent with reports in the literature for pyrolysis and steam reforming processes, in which the decrease in C_2_–C_4_ contents occurs with increasing temperature. The increase in CO production, significant in absolute terms but considerably lower than that of H_2_ in relative terms, suggests that CO production originates from the reforming reactions (R3, R4, and R5) and is partially offset by the progress of the WGS reaction (R6). It should be noted that, in polymer pyrolysis and catalytic steam reforming, cracking of C_2_–C_4_ and enhanced production of H_2_ and CO are usually observed between 700 °C and 800 °C [28,30,31]. Thus, the shift to the 900 °C range in the present study is, therefore, attributable to the absence of a catalyst. Likewise, the persistence of CH_4_ in the effluent gas provides a direct measure of the potential gain attributable to the introduction of a catalyst. Although Figure 2d suggests a moderate and progressive reduction in the methane content with the temperature increase from 800 °C to 900 °C and across all S/C increments, the behavior expected for the methane steam reforming reaction (R5), the reaction remains limited by the strong C–H bond in the symmetric molecular structure of methane, with a bond dissociation energy of 440 kJ·mol^−1^ [32].

The analysis of the effect of the steam-to-carbon ratio at a fixed temperature of 900 °C suggests that the pronounced gains in H_2_ yield occurred between S/C ratios of 2.5 and 7, above which further increases in the steam-to-carbon ratio did not result in substantial additional gains in hydrogen production. This behavior is consistent with a progressive reduction in steam conversion efficiency, in which methane and the light hydrocarbons are already largely consumed, so that each additional steam increment no longer contributes to hydrogen production. This same finding is recurrent in the steam reforming literature [9,33,34,35] and is here observed between S/C ratios of 7 and 12. Additionally, Figure 2b shows a moderate increase in CO between S/C 2.5 and 7, suggesting that the reforming reactions (R3, R4, and R5) remain incomplete in the absence of a catalyst, such that the CO supply from the reforming reactions exceeds the consumption promoted by the WGS reaction, followed by a marginal decline between S/C 7 and 12, suggesting saturation of the hydrocarbon reforming reaction and CO consumption by the WGS reaction.

The H_2_/CO ratio constitutes the main criterion for the suitability of the syngas for different valorization routes, since each application has its own stoichiometric requirements. Among the conditions investigated in this study (Figure 2c), the H_2_/CO ratio at 900 °C with S/C 7 approaches the range required by methanol synthesis (2:1) in the absence of CO_2_ and by the Fischer–Tropsch route with Fe- or Co- catalysts (from 2:1 to 2.2:1) [36]. It should be noted, however, that the H_2_/CO ratio alone does not constitute a sufficient suitability criterion, since it disregards the contents of the other gas components.

### 2.3. Footwear Waste Char Characteristics and Carbon Dioxide Adsorption Capacity

The amount of char collected in the pyrolysis and pyrolysis coupled with steam reforming experiments of this study ranged from around 33.83% to 38.54%. A basic mass balance of pyrolysis and pyrolysis coupled with steam reforming experiments is provided in Table S1 (Supplementary Materials). The physicochemical properties of the produced char are presented in Table 3 through proximate and ultimate analyses.

**Figure 3:**
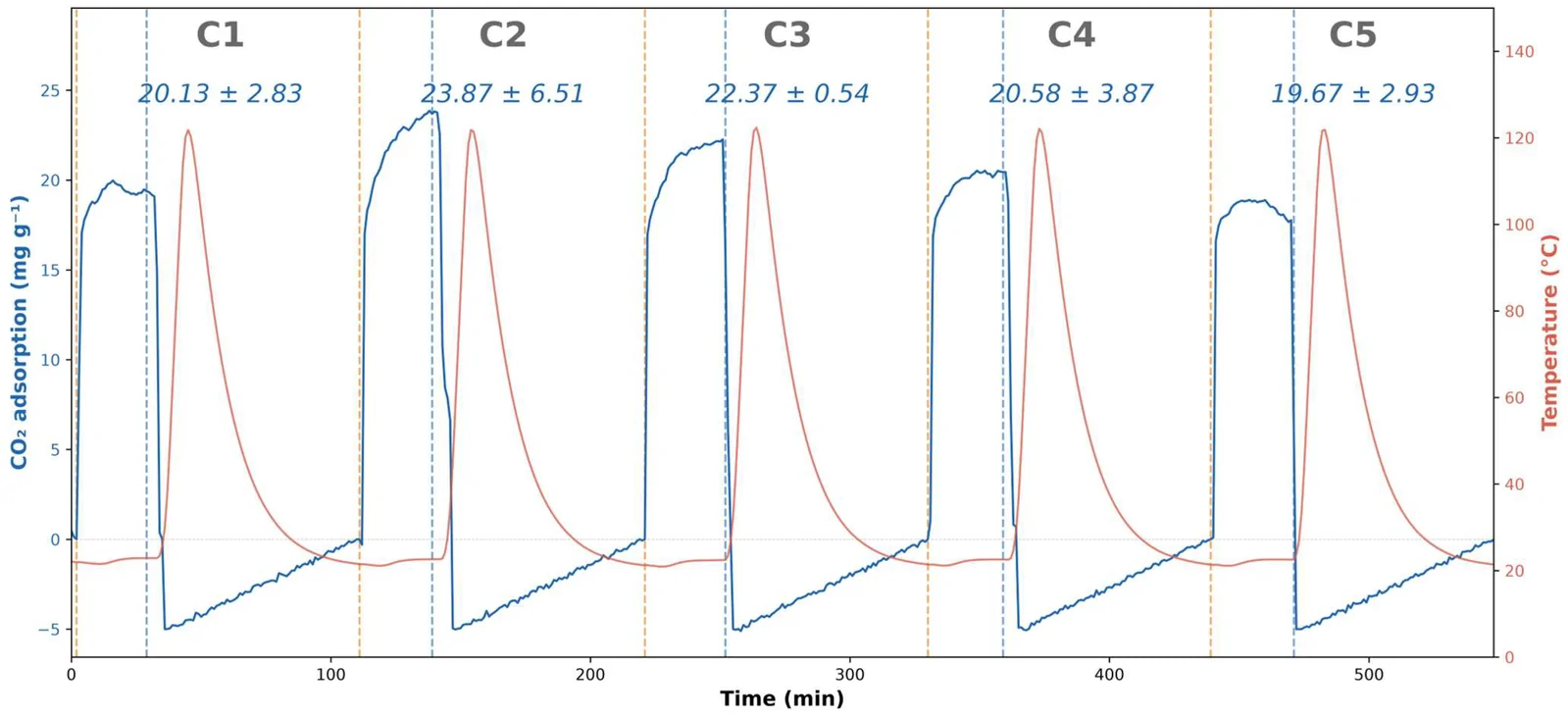
Footwear waste char CO_2_ adsorption cycles from triplicate measurements with standard deviations. The blue line represents the CO_2_ adsorption capacity (mg g^−1^), while the red line represents the temperature.

The CO_2_ adsorption capacity of the char produced from the pyrolysis of footwear waste at 600 °C was tested at 25 °C and atmospheric pressure. The results of the triplicate measurements are shown in Figure 3.

The char exhibited a CO_2_ adsorption capacity of 20.13 ± 2.83 mg g^−1^; each of the triplicate measurements is provided in Figure S2 (Supplementary Materials), consistent with chars derived from a mixture of post-consumer plastics produced by [37] at lower temperatures (10.4 mg g^−1^ at 450 °C and 13.6 mg g^−1^ at 500 °C). Likewise, the char from a nonrecyclable mixture of plastics from the rejected fraction of plastics collected in municipal solid waste, produced at 500 °C by [38], reached 6.2 mg g^−1^. Also, the char demonstrated stability in CO_2_ adsorption–desorption, maintaining its adsorption capacity over 5 consecutive cycles. Similar cyclic stability, with the adsorption capacity maintained over 5 cycles, was reported by [39] using H_3_PO_4_-activated biochar, ref. [40] using biochar activated with CO_2_ and K_2_CO_3_, and [41], who reported 98.5% regeneration over 5 cycles with a biochar.

Regarding the fundamental textural properties for CO_2_ adsorption, they are presented in Table 4, providing the necessary basis for interpreting the material’s adsorptive performance.

The char produced in this study exhibited a specific surface area (S_BET_) of 86.47 m^2^ g^−1^ and a pore volume of 0.123 cm^3^ g^−1^, values substantially higher than those reported by [37] for a different feedstock pyrolyzed at 450 °C (S_BET_ = 3.3 m^2^ g^−1^; V_p_ = 0.015 cm^3^ g^−1^) and at 500 °C (S_BET_ = 2.1 m^2^ g^−1^; V_p_ = 0.009 cm^3^ g^−1^), as well as to the char obtained by [38] at 500 °C (S_BET_ = 14.7 m^2^ g^−1^; V_p_ = 0.025 cm^3^ g^−1^). While the higher pyrolysis temperature employed in this study (600 °C) may have contributed to the enhanced textural development, it is important to note that differences in feedstock composition and other pyrolysis conditions may also play significant roles, and the individual effect of temperature cannot be isolated from cross-study comparisons. The surface area and pore volume in the footwear waste char were reflected in higher CO_2_ adsorption compared to the char of [37] (10.4 and 13.6 mg g^−1^) and [38] (6.2 mg g^−1^), suggesting higher pore development and availability of physical adsorption sites.

Although it should be noted that the CO_2_ uptake in this study, obtained for an as-pyrolyzed char without any post-synthesis physical or chemical activation step, corresponds to approximately 18% of the CO_2_ uptake reported for benchmark commercial activated carbons such as Norit RB2, 2.5 mmol g^−1^ (approximately 110.02 mg g^−1^) at 25 °C and 1 bar [42].

**Figure 4:**
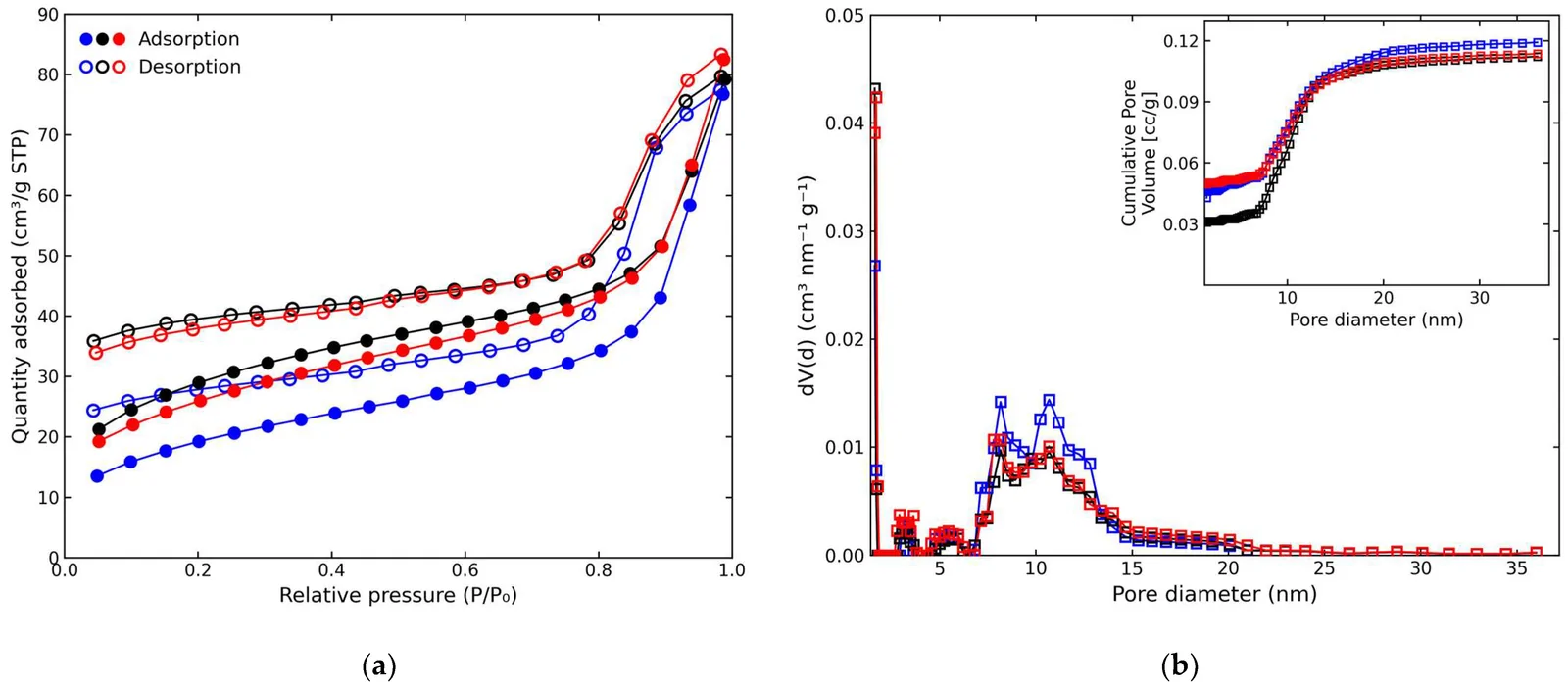
(**a**) Footwear waste char N_2_ adsorption-desorption isotherms in triplicate. (**b**) Pore size distribution and cumulative pore volume by DFT in triplicate.

Figure 4 shows the N_2_ adsorption–desorption isotherms measured at 77 K (a) together with the DFT pore-size distributions and the cumulative pore-volume inset, obtained for three independent replicates of the char produced at 600 °C. The replicates display the same shape and are correlated over the entire pressure range, which attests to the reproducibility of both the material and the physisorption measurements.

The coexistence of an uptake at P/P_0_ ≈ 0.05 in the N_2_ adsorption, characteristic of micropore filling (IUPAC type-I contribution) [43], a gradual increase at intermediate pressures and an upturn above P/P_0_ ≈ 0.8 together with a hysteresis loop, characteristic of mesopore presence (IUPAC type-IV(a)) [43], identifies the isotherms as micro-mesoporous. The incomplete closure of the desorption curve at low P/P_0_ is a feature commonly reported for solids containing narrow micropores [43], attributed to slow re-equilibration and to adsorbate retained in the smallest pores. The pore size distribution rationalizes this argument with a narrow peak at the start reflecting microporosity and a broad mesopore centered at ≈8 nm.

The footwear waste char produced exhibits CO_2_ adsorption consistent with the textural analysis, with uptake of CO_2_ in the narrow micropores and less sustained by the weaker adsorption potential for CO_2_ molecules in the mesopores or wider pores [44]. In other words, the surface generated at 600 °C is effective for CO_2_ adsorption but limited by the microporosity. This identifies the micropore volume as the parameter to target for enhancing the CO_2_ adsorption capacity, mainly through physical or chemical activation, indicating potential for this waste-derived material as a low-cost precursor for CO_2_ sorbent production after further treatment.

## 3. Material and Methods

### 3.1. Footwear Waste Obtaining and Characterization

The mixed plastic footwear waste was collected from Ambiente Verde Indústria Ltd.a., located in Taquara, Rio Grande do Sul, Brazil. A single batch of approximately 20 kg was sampled to ensure representativeness, since batch-to-batch variability is expected in the supplied waste. According to the company, the main composition includes blends of polyesters (~65%) > polyurethane (PU) (~25%) > ethylene-vinyl acetate (EVA) (~6%) > cotton (~4%). The collected sample (~20 kg) was homogenized and reduced to ca. 500 g by repeated coning and quartering. At each step, the sample was divided into four clockwise-numbered quadrants; two opposite quarters (alternating 1–3 and 2–4 between cycles) were retained and remixed, while the remaining two were discarded, until a representative subsample of 500 g was obtained and used for the study. The samples were used as received in this study. A representative image of the material is provided in Figure S1 (Supplementary Materials).

The proximate analysis (moisture, volatile matter, ash content, and fixed carbon) was determined according to ref. [45]. All analyses were performed in triplicate. Fixed carbon was calculated by difference. The ultimate analysis (C, H, N, and S) was conducted according to ref. [46] and [47], while the oxygen content was calculated by difference. The thermal stability of the samples was analyzed by thermogravimetric analysis (TGA) (STA 449 F3 Jupiter, Netzsch, Selb, Germany), using 10 mg of sample, a heating rate of 5 °C min^−1^ from ambient temperature to 900 °C, and a nitrogen flow rate of 50 mL min^−1^.

### 3.2. Pyrolysis and Steam Reforming Process

**Figure 5:**
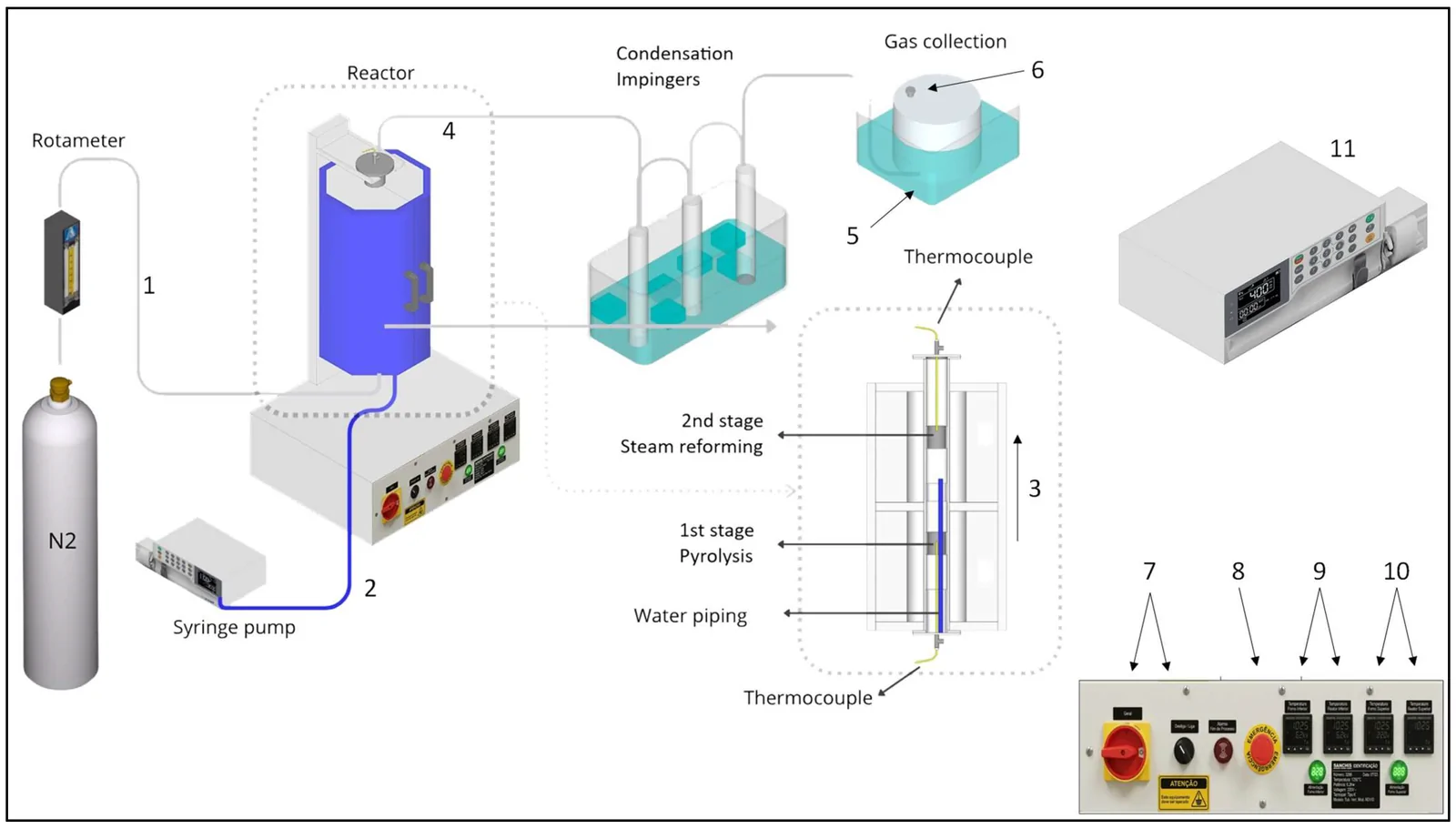
Schematic diagram of the proof-of-concept setup for integrated valorization of mixed footwear waste. (1) N_2_ Carrier gas. (2) Water feed; (3) Pyrolysis volatiles; (4) Reformer effluent; (5) U tube; (6) Sampling point (rubber septum); (7) Power buttons; (8) Emergency button; (9) First-stage temperature controller; (10) Second-stage temperature controller; (11) Syringe Pump.

The pyrolysis and steam reforming in a series reactor system is shown in Figure 5. The reactor consists of a single tubular stainless-steel vessel (60 mm outer diameter, 53 mm inner diameter, 600 mm total length, and 500 mm total heated length) divided into two independently heated isothermal stages in series.

In a typical experiment, approximately 2 g of footwear waste was placed in a steel crucible and pyrolyzed in the first-stage pyrolysis reactor, heated at 40 °C min^−1^ to a final temperature of 600 °C. All experiments were conducted at atmospheric pressure (~1 bar absolute), maintained under a slight positive overpressure of ~10 mbar to prevent ambient air ingress into the system. In the second-stage reforming reactor, the steel crucible was filled with only 2 g of quartz, as a blank, and heated to 700 °C, 800 °C, or 900 °C. When the temperature of the second-stage reforming reactor reached the target reforming temperature (700, 800, or 900 °C), the first-stage pyrolysis reactor was activated. Water was introduced into the second-stage steam reforming reactor via a syringe pump once the first-stage reactor reached 190 °C, ensuring that steam was present in the reformer before significant volatile release (see TGA profile in Figure 1). Liquid water was fed to the system at a controlled mass flow rate of 10, 30 and 50 mL h^−1^ to achieve the target steam-to-carbon (S/C) ratios investigated of 2.5, 7, and 12, respectively. The steam-to-carbon ratio (S/C) was defined as: S/C = mol H_2_O fed in the reforming reactor / mol C in the footwear waste determined from the elemental composition of the raw waste. Nitrogen was used as the carrier gas at a flow rate of 25 mL min^−1^, and the produced gases were collected in a 4 L graduated beaker. All gas volume and yield values reported in this work were normalised to STP conditions (273.15 K, 101.325 kPa, molar volume = 22.41 L mol^−1^).

The gas samples were analyzed using a gas chromatograph (Master GC, Dani Instruments, Cologno Monzese, Italy) equipped with a thermal conductivity detector (TCD) coupled to a Carboxen 1006 capillary column (Supelco, Bellefonte, PA, USA) for the analysis of H_2_, CO, CH_4_, and CO_2_, and a flame ionization detector (FID) coupled to a PLOT Al_2_O_3_/KCl capillary column (Agilent, Santa Clara, CA, USA) for C_2_H_6_, C_2_H_4_, C_3_H_8_, C_3_H_6_, C_2_H_2_, C_3_H_4_, C_4_H_10_, and C_4_H_8_. Nitrogen was used as the carrier gas for both the TCD and the FID. All experiments were conducted in triplicate.

### 3.3. Footwear Waste Char Characterization and Carbon Dioxide Adsorption Capacity

The textural properties (surface area, pore volume, and pore size distribution) of the footwear waste char were determined using a Quantachrome Instruments Nova 1200e analyzer (Boynton Beach, FL, USA).

The footwear waste char was used as an adsorbent for CO_2_ capture. The CO_2_ adsorption tests were carried out at 25 °C and atmospheric pressure in thermogravimetric equipment (STA 449 F3 Jupiter, Netzsch, Selb, Germany), employing 10 mg of char, following the same methodology reported by [39] and [41]. When the adsorbent saturation point was reached, the CO_2_ flow (50 mL min^−1^) was interrupted, and the sample was heated to 120 °C under N_2_ flow (50 mL min^−1^) to promote desorption. The procedure was repeated until five complete adsorption–desorption cycles were performed.

### 3.4. Graphical Abstract

ChatGPT (GPT-5, OpenAI) was used to assist in the preparation of the graphical abstract. The tool was used solely for generating the graphical elements of the graphical abstract. The authors reviewed, edited, and finalized the graphical abstract and take full responsibility for its content.

## 4. Conclusions

A possible route to valorize footwear waste was studied regarding the production of hydrogen-rich syngas and char for CO_2_ adsorption.

Steam reforming at 700 °C contributed to a slight increase in production of H_2_ and CO compared to the pyrolysis-only experiment. At this temperature the system lies below the thermal activation threshold of the reforming reactions, a condition in which gas production remains low and independent of the steam-to-carbon ratio.

In the transition to 800 °C, the system remains kinetically limited, with a balance between the formation and consumption of intermediates and only slight progress in the overall conversion.

In the transition to 900 °C, the reforming reactions become kinetically dominant, promoting the pronounced consumption of C_2_–C_4_ and the substantial release of hydrogen, whereas the CO distribution becomes modulated by the progress of the WGS reaction. Residual concentrations of methane and light hydrocarbons still persist in the exhaust gas and could be further reduced by adding a catalyst.

The steam-to-carbon ratio analysis suggests that, at 900 °C, the pronounced gains in H_2_ yield occurred between S/C of 2.5 and 7, above which further increases in the steam-to-carbon ratio did not result in substantial additional gains in hydrogen production.

From the standpoint of product gas utilization, the condition of 900 °C with S/C 7 provides an H_2_/CO ratio compatible with catalytic synthesis routes, although still requiring the prior removal of the other gas components to meet the stoichiometric requirements.

The footwear waste char produced exhibits a CO_2_ adsorption capacity (20.13 ± 2.85 mg g^−1^) consistent with the textural analysis and is effective for CO_2_ adsorption but is limited by the microporosity. This identifies the micropore volume as the parameter to target for enhancing the CO_2_ adsorption capacity, mainly through physical or chemical activation, indicating the potential of this waste-derived material as a low-cost precursor for CO_2_ sorbent production after further treatment.

## Figures and Tables

**Table 1:** Proximate analysis and elemental composition of the footwear waste.

Proximate Analysis ^1^	%wt.	Elemental Composition ^1^	%wt.
Moisture	0.35 ± 0.05	C	58.42 ± 1.72
Ash	7.5 ± 0.21	H	6.63 ± 0.61
Fixed carbon ^2^	13.9 ± 0.54	N	1.43 ± 0.13
Volatile matter	78.6 ± 0.32	S	0.11 ± 0.01
		O ^2^	25.91 ± 2.21

^1^ Dry basis, ^2^ By difference.

**Table 2:** Main reactions expected in the pyrolysis and steam reforming of footwear waste.

Reaction	Name of Reaction	Reactions
R1	Pyrolysis	Footwear waste → C_n_H_m_ + C_n_H_m_O_x_ + H_2_ + CO + CH_4_ + CO_2_ + Char
R2	Volatiles thermal cracking	C_n_H_m_O_x_ → C_a_H_b_O_c_ + C_p_H_q_ + H_2_ + CO + CH_4_ + CO_2_ + C_(s)_
R3	Hydrocarbon reforming	C_n_H_m_ + nH_2_O ⇄ nCO + [n + (m/2)] H_2_
R4	Oxygenated hydrocarbons reforming	C_n_H_m_O_x_ + (n − x) H_2_O ⇄ nCO + [n − x + (m/2)] H_2_
R5	Methane reforming	CH_4_ + H_2_O ⇄ CO + 3H_2_
R6	Water-gas shift (WGS)	CO + H_2_O ⇄ CO_2_ + H_2_
R7	Boudouard	CO_2_ + C ⇄ 2CO

**Table 3:** Proximate analysis and elemental composition of the footwear waste char.

Proximate Analysis ^1^	%wt.	Ultimate Analysis ^1^	%wt.
Moisture	0.26 ± 0.15	C	59.35 ± 2.28
Ash	36.3 ± 0.4	H	1.60 ± 0.21
Fixed carbon ^2^	35.1 ± 0.8	N	1.21 ± 0.04
Volatile matter	28.6 ± 0.4	S	0.17 ± 0.02
		O ^2^	1.37 ± 2.93
		H/C atomic ratio ^3^	0.32 ± 0.04
		O/C atomic ratio ^3^	0.02 ± 0.04

^1^ Dry basis. ^2^ By difference. ^3^ Atomic ratio calculated from the elemental composition (mass fractions) using atomic masses: C = 12.01 g mol^−1^, H = 1.008 g mol^−1^, and O = 16.00 g mol^−1^; Oxygen content was calculated by difference; the elevated uncertainty reflects the low residual Oxygen concentration in this high-ash char produced at 600 °C.

**Table 4:** Textural properties of footwear waste char.

Surface Area BET S_BET_ (m^2^ g^−1^)	Total Pore Volume V_p_ (cm^3^ g^−1^)	Micropores Volume V_m_ (cm^3^ g^−1^)	Mesopore Volume V_p-m_ (cm^3^ g^−1^)	Average Pore Size (nm)
86.47 ± 16.49	0.1227 ± 0.004	0.0427 ± 0.0099	0.08 ± 0.0085	5.82 ± 1.05

## Data Availability

Data available upon reasonable request.

## Supplementary Materials

The following supporting information can be downloaded at: https://www.mdpi.com/article/10.3390/molecules31193490/s1, Figure S1: Footwear waste used in this study; Figure S2: Footwear waste char CO_2_ adsorption cycles in triplicate measurements (a), (b), (c); Table S1: Basic mass balance from pyrolysis and pyrolysis steam reforming of footwear waste experiments.
