# Supplementary material for: Pyrolysis and Steam Reforming in Series of Footwear Waste to Hydrogen-Rich Syngas and Char for CO2 Adsorption
Source: Molecules. 2026 Oct 1;31(19):3490. doi: 10.3390/molecules31193490 (PMC13666703; doi:10.3390/molecules31193490)
Supplement: Supplementary file 1 [file molecules-31-03490-s001.zip › molecules-4505574-supplementary.pdf]

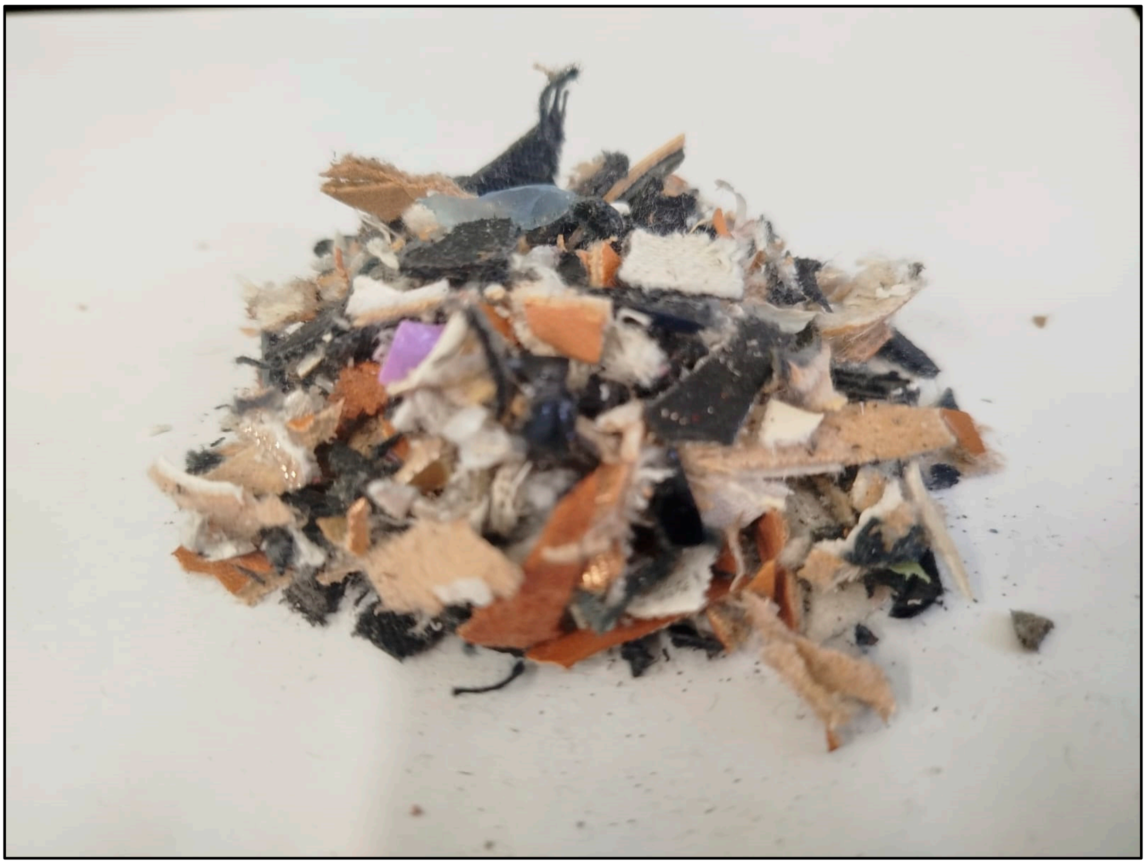

Figure S1. Footwear waste used in the study.

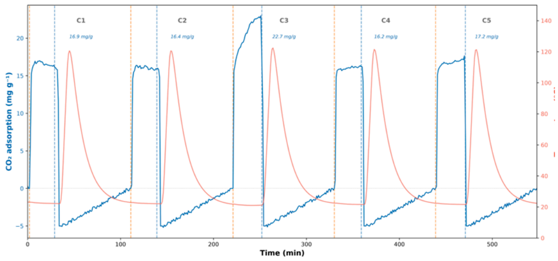

(**a**)

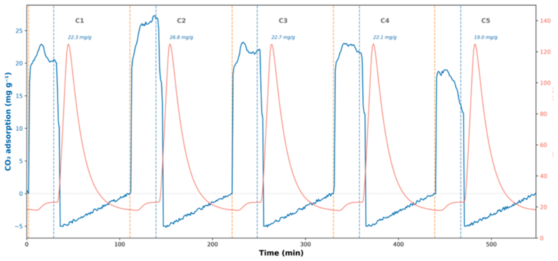

(**b**)

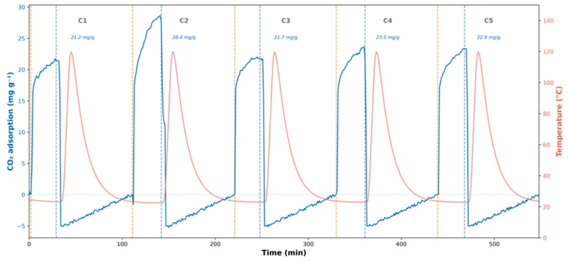

(c)

Figure S2. Footwear waste char CO_2 adsorption cycles in triplicate measurements (a), (b), (c).

Table S1. Basic mass balance from pyrolysis and pyrolysis steam reforming of footwear waste experiments.

**Pyrolysis temperature (°C)**	600	600	600	600	600	600	600	600	600	600
**Steam Reforming Temperature (°C)**	-	700	700	700	800	800	800	900	900	900
**Steam-to-carbon ratio**	-	2.5	7	12	2.5	7	12	2.5	7	12
**Gas yield (%wt.)**	10.70 ± 4.18[1]	13.69 ± 5.97	9.41 ± 3.55	10.18 ± 4.81	16.65 ± 4.53	18.06 ± 9.50	30.44 ± 5.97	17.77 ± 4.92	35.28 ± 3.92	39.93 ± 4.39
**Char yield (%wt.)**	38.54 ± 2.92	35.78 ± 1.50	33.83 ± 0.85	35.36 ± 1.59	35.28 ± 2.29	35.94 ± 0.39	34.71 ± 0.68	37.31 ± 1.20	36.08 ± 1.41	37.26 ± 3.10
**Oil yield (%wt.)**	50.76 ± 5.35	50.53 ± 4.48[1]	56.76 ± 3.78[1]	54.46 ± 5.97[1]	48.07 ± 6.77[1]	46.00 ± 9.86[1]	34.85 ± 5.98[1]	44.92 ± 4.02[1]	28.64 ± 5.29[1]	22.81 ± 7.47[1]

[1] By diference; ± Standard deviation of data sample.
